# Combination of metformin and parthenolide inhibits breast cancer in mice by apoptosis induction and angiogenesis inhibition

**DOI:** 10.3389/fimmu.2026.1843223

**Published:** 2026-06-10

**Authors:** Raghad K. Alomari, Moudi M Alasmari, Heba K Alshaeri, Alexandra Makai, Wamidh H Talib, Márta Hock

**Affiliations:** 1 Faculty of Pharmacy, Applied Science Private University, Amman, Jordan; 2 College of Medicine, King Saud bin Abdulaziz University for Health Sciences (KSAU-HS), Jeddah, Saudi Arabia; 3 King Abdullah International Medical Research Centre (KAIMRC), Jeddah, Saudi Arabia; 4 Department of Clinical Pharmacology, Faculty of Medicine, King Abdulaziz University, Rabigh, Saudi Arabia; 5 Institute of Physiotherapy and Sports Science, Faculty of Health Sciences, University of Pécs, Pécs, Hungary; 6 Physical Activity Research Group, János Szentágothai Research Center, University of Pécs, Pécs, Hungary; 7 Faculty of Allied Medical Sciences, Applied Science Private University, Amman, Jordan

**Keywords:** alternative therapy, antitumor, combination anticancer therapies, natural products, VEGF

## Abstract

**Background:**

Metformin (MET), a widely used antidiabetic agent, and parthenolide (PAR), a natural compound recognized for its anti-inflammatory properties, have both been extensively explored for their anticancer potential. The present study aimed to investigate the anticancer activity of their combination against breast cancer *in vitro* and *in vivo*.

**Methods:**

The antiproliferative effects of MET, PAR, and their combination were evaluated against a panel of breast cancer cell lines (EMT6/P, MDA-MB-231, and T47D) using the MTT assay. Synergistic interactions were assessed using the isobolographic method to calculate the combination index (CI). Apoptosis and angiogenesis were further investigated in T47D cells through caspase-3 activity assays and measurement of vascular endothelial growth factor (VEGF) levels, respectively. For *in vivo* assessment, EMT6/P cells were inoculated in BALB/c mice, and the antitumor effects of MET, PAR, and their combination were evaluated by monitoring tumor size. Treatment-related toxicity was assessed by measuring serum levels of aspartate transaminase (AST), alanine transaminase (ALT), and creatinine.

**Results:**

MET and PAR exhibited a clear synergistic antiproliferative interaction against all tested breast cancer cell lines, with varying degrees of synergism observed. *In vivo*, combination therapy produced a significant reduction in tumor size, with a cure rate of 50% and zero treatment-related mortality. The combination significantly induced caspase-3-dependent apoptosis and reduced VEGF expression compared to either agent alone. Serum AST, ALT, and creatinine levels in combination-treated tumor-bearing mice remained within normal ranges, supporting the safety of this regimen.

**Conclusion:**

The combination of MET and PAR demonstrates synergistic anticancer activity against breast cancer both *in vitro* and *in vivo*, acting through apoptosis induction and angiogenesis inhibition. Given these promising findings, further studies are warranted to provide more comprehensive molecular and mechanistic characterization of this combination and to support its future clinical evaluation.

## Introduction

Cancer remains a critical global health challenge and continues to be among the leading causes of mortality worldwide (1). In 2022, approximately 20 million new cancer cases and nearly 10 million cancer-related deaths were reported globally. It is anticipated that by 2050, there will be a 77% increase in new cases, potentially reaching around 35 million. Among various malignancies, breast cancer is one of the most prevalent, accounting for 11.5% of all newly diagnosed cancer cases and 6.8% of cancer-related deaths worldwide in 2022 (2). Despite the availability of conventional cancer treatments such as surgery, chemotherapy, and radiotherapy, their clinical efficacy is often compromised by significant limitations, including the development of cellular resistance and substantial systemic toxicity (3). Given the rapidly increasing global cancer burden, it is essential to intensify our efforts to combat this disease by developing new, cost-effective treatment strategies that yield meaningful clinical outcomes (2).

**Figure 1:**
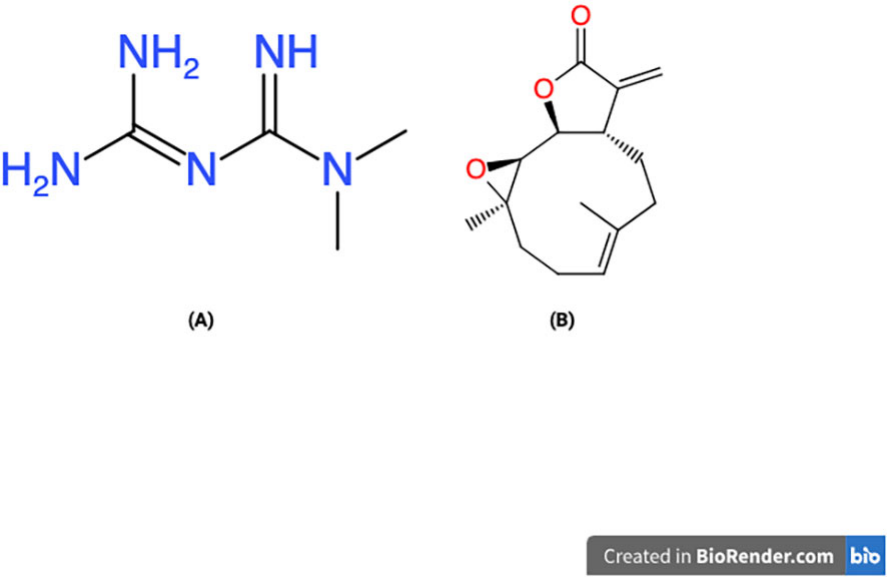
Chemical structures of **(A)** metformin and **(B)** parthenolide.

The use of metformin (MET), a first-line antidiabetic agent, can be traced back to the historical application of extracts from French lilac (*Galega officinalis*) (4). This plant, identified in the 19th century as rich in guanidine, was utilized for its ability to lower blood glucose levels (5). Metformin (1,1-dimethylbiguanide) is a synthetic biguanide formed by combining two guanidine units into a single molecule (4) (**Figure 1A**). This orally effective antidiabetic drug, synthesized in the 1920s, has become the most prescribed hypoglycemic medication for type 2 diabetes mellitus due to its safety and efficacy (6). MET reduces blood glucose by decreasing hepatic gluconeogenesis and enhancing insulin sensitivity, which increases peripheral glucose uptake and utilization, thereby lowering plasma glucose levels (7). Recent research has found that MET alters the levels of proteins involved in various cellular processes. This has led to increased interest in studying MET for multiple diseases, thus expanding its potential clinical applications beyond its antidiabetic effects. It has been shown to significantly impact various conditions, including cardiovascular diseases (CVDs), liver diseases, obesity, neurodegenerative diseases such as cognitive dysfunction and dementia, and renal diseases (8). Researchers have also explored repurposing MET for the treatment of polycystic ovary syndrome (PCOS), and rheumatoid arthritis, as an anti-ageing drug, in treating parasitic infections like malaria, as an antibiotic, and for managing coronavirus disease (COVID-19) (4). Most notably, numerous preclinical and clinical studies have investigated MET, both as a monotherapy and in combination with other agents, for its potential in cancer prevention and treatment (7). MET has demonstrated several advantages across multiple cancer types, including breast, endometrial, lung, blood, colorectal, melanoma, and bone cancers (6–8). Some of these benefits include suppression of cancer cell growth, survival, and metastasis, induction of apoptosis and autophagy, modulation of the tumor microenvironment, suppression of cancer stem-cell activity, regulation of epigenetic modifications, and mitochondrial energy metabolism regulation (6, 8). Additionally, MET exhibits anti-angiogenic and immunoregulatory effects, all of which are associated with improved overall survival outcomes in cancer patients (6).

On the other hand, parthenolide (PAR) is a sesquiterpene lactone extracted from the feverfew plant (*Tanacetum parthenium*). The name “feverfew” is derived from the Latin term “febrifugia,” meaning “fever reducer,” indicating one of the primary pharmacological benefits of this plant, its anti-inflammatory properties (9). PAR is reported to be the most biologically active constituent in the leaves and flowers of feverfew and is considered responsible for its therapeutic effects (9, 10). This compound is known for its remarkable anti-inflammatory effects and has historically been used to address conditions like high fever, headaches, rheumatoid arthritis, and menstrual cramps (11). It is now recommended for use in the prevention of migraines and management of conditions such as endometriosis, cystic fibrosis, rheumatoid arthritis, and gout (12, 13). The notable anti-inflammatory effects of PAR have raised the possibility of its application in cancer treatment. Its antitumor properties were first identified in 1973, and it was subsequently patented for cancer inhibition in 2005 (9, 14). Extensive research has since explored its anticancer properties, demonstrating its efficacy across a variety of cancers, including breast, skin, colon, prostate, lung, cervix, and numerous blood cancers (14). The structural characteristics of PAR enable it to impact essential biological functions, including cell signaling pathways, cellular proliferation, and apoptosis. In particular, the presence of the α-methylene-γ-lactone moiety (**Figure 1B**), a key structural feature of sesquiterpene lactones, allows PAR to alkylate various intracellular nucleophiles such as L-cysteine, glutathione, and thiol-containing proteins, leading to the formation of adducts that may contribute to its cytotoxic effects (9). Despite this reactive structural feature, PAR has been reported to exhibit selective anticancer activity by modulating signaling pathways that preferentially target tumor cells, including cancer stem cells, while largely sparing normal stem cells (14).

Given the heterogeneity and multifactorial nature of cancer, relying on monotherapy for treating and completely eradicating different cancer types is increasingly seen as an ineffective strategy (15). This has led to the development of combination therapies, which have demonstrated improved efficacy in cancer treatment by simultaneously targeting multiple molecular pathways critical for cancer cell survival and the development of adaptive resistance (16, 17). The use of a combination of two or more therapeutic agents is fundamental in cancer treatment, offering significant advantages such as enhanced efficacy, better response rates, reduced resistance, and lower toxicity (15, 17). MET and PAR, administered alongside other therapeutic agents, have shown substantial antitumor potential in both *in vitro* and *in vivo* models (14, 18). However, to date, their combined use has not been explored. MET has been widely reported to exert anti-proliferative effects primarily through modulation of cellular metabolism and inhibition of the phosphoinositide 3-kinase/protein kinase B/mechanistic target of rapamycin (PI3K/Akt/mTOR) pathway (6). In contrast, PAR is known for its pro-apoptotic and anti-inflammatory effects, particularly through inhibition of nuclear factor kappa B (NF-κB) signaling and induction of oxidative stress (14). Their distinct mechanisms of action suggest a potentially complementary effect, as simultaneous targeting of metabolic regulation and apoptotic signaling pathways may enhance therapeutic efficacy beyond that of single-agent treatments.

In the present work, we examined the combined effect of MET and PAR in a breast cancer mouse model. We hypothesized that their co-administration would produce synergistic effects through the integration of their diverse molecular actions.

## Materials and methods

### Chemicals, cell lines and culture conditions

Metformin was obtained from Wanbury Ltd. (Navi Mumbai, India), and parthenolide was sourced from MedChemExpress (Princeton, NJ, USA). The EMT6/P (mouse mammary), MDA-MB-231, and T47D (human breast carcinoma) cell lines, along with the Vero (monkey kidney) normal cell line, were purchased from the European Collection of Cell Cultures (ECACC). EMT6/P and Vero cells were cultured in Minimum Essential Medium (MEM), MDA-MB-231 cells in Dulbecco’s Modified Eagle’s Medium (DMEM), and T47D cells in Roswell Park Memorial Institute (RPMI)-1640 medium. All media were supplemented with 10% fetal calf serum (Corning, Mediatech Inc., Manassas, VA, USA), 1% L-glutamine (Servicebio, Wuhan, China), 1% penicillin-streptomycin (Eurobio Scientific, Les Ulis, France), and 0.1% gentamycin (Biowest, Nuaillé, France). Cells were incubated at 37 °C in 5% CO_2_ and 95% humidity.

### MTT cell viability assay

Freshly cultured cancer cells were harvested, washed, and resuspended in complete growth medium. Cell viability was assessed using trypan blue exclusion. Cells were seeded into 96-well flat-bottom tissue culture plates at a density of 1 × 10^4^ cells per well in 100 μL of medium. After overnight incubation, cells were treated in triplicate with serial dilutions of MET (180–1.4 mM) (19, 20), PAR (20–0.16 μM) (21), or their combinations, in which one agent was applied across a range of concentrations while the second agent was maintained at a fixed concentration, and vice versa, as previously described (22) (**Table 1**), yielding a final volume of 200 μL per well. Following 48 hours of treatment, cell viability was assessed using the 3-(4,5-dimethylthiazol-2-yl)-2,5-diphenyltetrazolium bromide (MTT) assay. For this, 100 μL of media was aspirated from each well and replaced with 20 μL of MTT solution, followed by incubation at 37 °C for 3 h. Subsequently, 100 μL of dimethylsulfoxide (DMSO) (Guangdong Guanghua Sci-Tech Co., Ltd., Shantou, China) was added to each well to solubilize the formazan crystals, and plates were incubated for an additional hour. Absorbance was measured at 550 nm using a microplate reader (BioTek, USA; Model No. EL-x800). Untreated cells served as negative controls, while cells treated with doxorubicin HCl (Ebewe Pharma GmbH Nfg. KG, Unterach, Austria) were included as a positive control. Cell viability, expressed as percentage survival rate relative to untreated controls, was calculated using the following formula:

**Table 1:** Single-agent IC_50_ values and combination treatment concentrations of MET (mM) and PAR (µM) expressed as absolute concentrations and fractions of IC_50_ values across *in vitro* cell lines.

Cell line	MET IC_50_	PAR IC_50_	Combination (absolute concentrations)	Combination (fractions of IC_50_)
EMT6/P	32.46	8.8	MET (0.16, 0.31, 0.63, 1.25, 2.5, 5, 10, 20) + PAR (4); PAR (0.03, 0.06, 0.13, 0.25, 0.5, 1, 2, 4) + MET (20)	MET (0.005, 0.01, 0.02, 0.04, 0.08, 0.15, 0.31, 0.62) + PAR (0.45); PAR (0.003, 0.007, 0.015, 0.03, 0.06.0.11, 0.23, 0.45) + MET (0.62)
MDA-MB-231	181.59	>20	MET (0.7, 1.41, 2.81, 5.63, 11.25, 22.5, 45, 90) + PAR (10); PAR (0.08, 0.16, 0.31, 0.63, 1.25, 2.5, 5, 10) + MET (90)	MET (0.004, 0.008, 0.02, 0.03, 0.06, 0.12, 0.25, 0.5) + PAR (0.5); PAR (0.004, 0.008, 0.02, 0.03, 0.06, 0.12, 0.25, 0.5) + MET (0.5)
T47D	16.26	1.72	MET (0.06, 0.13, 0.25, 0.5, 1, 2, 4, 8) + PAR (1); PAR (0.01, 0.02, 0.03, 0.06, 0.13, 0.25, 0.5, 1) + MET (8)	MET (0.004, 0.008, 0.015, 0.03, 0.06, 0.12, 0.25, 0.49) + PAR (0.58); PAR (0.006, 0.012, 0.017, 0.03, 0.08, 0.15, 0.29, 0.58) + MET (0.49)
Vero	61.19	>20	MET (0.23, 0.47, 0.94, 1.88, 3.75, 7.5, 15, 30) + PAR (10); PAR (0.08, 0.16, 0.31, 0.63, 1.25, 2.5, 5, 10) + MET (30)	MET (0.004, 0.008, 0.015, 0.03, 0.06, 0.12, 0.25, 0.49) + PAR (0.5); PAR (0.004, 0.008, 0.02, 0.03, 0.06, 0.12, 0.25, 0.5) + MET (0.49)

Values in the “Combination (Fractions of IC_50_)” column are based on serial dilutions initiated at approximately one-half of the corresponding single-agent IC_50_ value, with concentrations rounded to practical experimental values.


$$\text{Cell survival rate }\left(\%\right)=\left(OD\text{ of treated cells}/OD\text{ of untreated controls}\right)\times 100.$$

### Calculation of combination index

The half-maximal inhibitory concentration (IC_50_) is defined as the concentration of a drug needed for 50% inhibition or killing of cells compared to the untreated cells. A nonlinear regression test was applied to the data to obtain IC_50_ values for single and combination treatments. The isobolographic method was employed to assess the nature of the interaction between MET and PAR. The combination index (CI) was calculated using the following equation and results were interpreted as described below (23):


$$\text{CI}=\left(D\right)1/\left(Dx\right)1+\left(D\right)2/\left(Dx\right)2+\alpha \left(D\right)1\left(D\right)2/\left(Dx\right)1\left(Dx\right)2$$

where (*Dx*)1 = IC_50_ of drug 1 (MET) alone; (*D*)1 = IC_50_ of drug 1 (MET) in combination with drug 2 (PAR); (*Dx*)2 = IC_50_ of drug 2 (PAR) alone; (*D*)2 = IC_50_ of drug 2 (PAR) in combination with drug 1 (MET); α = 0 for mutually exclusive or 1 for mutually nonexclusive modes of drug action.

Interpretation of results: antagonism for CI value above 1.3; moderate antagonism for CI value of 1.1 to 1.3; additive interaction for CI value of 0.9 to 1.1; slight synergism for CI value of 0.8 to 0.9; moderate synergism for CI value of 0.6 to 0.8; synergism for CI value of 0.4 to 0.6; and strong synergism for CI value of 0.2 to 0.4.

### Induction of Caspase-3 activity in T47D cells

After 48 hours of treatment with 7 mM MET, 0.2 μM PAR, their combination, or media alone (negative control), cells were resuspended in phosphate-Buffered Saline (PBS) to a final concentration of 100 cells/ml and subjected to repeated freeze-thaw cycles to lyse the cells and release intracellular contents. The lysate was then centrifuged at 2000 rpm for 20 minutes to precipitate the cellular debris. The caspase-3 activity in the supernatant was measured according to the manufacturer’s instructions provided with the assay kit (Human Caspase-3 (CASP-3) ELISA Kit, catalogue no. SL2079Hu, Sunlong Biotech Co., Ltd, Hangzhou, China).

### Measuring vascular endothelial growth factor expression in T47D cells

T47D cells were cultured at a concentration of 1 × 10^5^ cells/ml and treated for 48 hours with one of the following treatments: 7 mM MET, 0.2 μM PAR, their combination, or media alone (negative control). Culture supernatants were aseptically collected and centrifuged at 2000 rpm for 20 minutes. The effect of these treatments on vascular endothelial growth factor (VEGF) expression was measured following the manufacturer’s instructions provided with the assay kit (Human VEGF ELISA kit, catalogue no. GW1811Hu, GenoChem world sl., Valencia, Spain).

### Anticancer therapy on experimental animals

All experimental procedures were conducted in accordance with standard ethical guidelines and were approved by the institutional review board (IRB) in the Faculty of Pharmacy - Applied Science Private University (approval no. 2025-PHA-27). The study utilized 24 female BALB/c mice (4–6 weeks old, weighing 21–25 g). Mice were kept in standard cages with wood shavings as bedding under controlled conditions: temperature maintained at approximately 25 °C, humidity at 50–60%, a 12-hour light/dark cycle, and continuous air ventilation.

Actively proliferating EMT6/P cells were harvested by trypsinization, centrifuged, washed, counted, and resuspended in MEM at a concentration of 1 × 10^6^ cells/mL. Cell viability was confirmed using the trypan blue exclusion method, and a tumorigenic dose of 1 × 10^5^ viable cells in 0.1 mL was injected subcutaneously into the abdominal region of each mouse. Tumors were allowed to grow for 14 days, after which tumor dimensions were measured using a digital caliper. Tumor volume was calculated using the formula: A × B² × 0.5, where A represents the longest diameter and B is the diameter perpendicular to A (24). Mice with established tumors were randomly divided into four groups (n = 6 per group), ensuring balanced average initial tumor volumes across groups. Group I received daily intraperitoneal (IP) injections of MET (80 mg/kg/day) in PBS (0.1 mL) (25); Group II received PAR (40 mg/kg/day) in olive oil (0.1 mL) IP (26); Group III received a combination of MET (80 mg/kg/day) and PAR (40 mg/kg/day) IP; and Group IV (control) received PBS and olive oil IP as vehicles. Treatments were administered for 7 consecutive days, after which tumor sizes were remeasured. The mice were then humanely euthanized by cervical dislocation in accordance with approved institutional ethical guidelines. Tumors were subsequently excised, weighed, and preserved in 10% formalin.

### Determination of aspartate transaminase, alanine transaminase, and creatinine serum levels

Hepatic and renal function parameters in all experimental mice were evaluated using commercially available diagnostic kits (DiaSys Diagnostic Systems GmbH, Holzheim, Germany) in accordance with the manufacturer’s instructions.

### Statistical analysis

All data are presented as mean ± standard error of the mean (SEM). Statistical differences among experimental groups were assessed using one-way analysis of variance (ANOVA) followed by an appropriate *post hoc* test. Statistical significance was set at *p* < 0.05. IC_50_ values for MET and/or PAR were determined via nonlinear regression analysis using the Statistical Package for the Social Sciences (SPSS Inc., Released 2020, PASW Statistics for Windows, Version 27.0, Chicago, IL, USA).

## Results

### Cytotoxic effects

**Figure 2:**
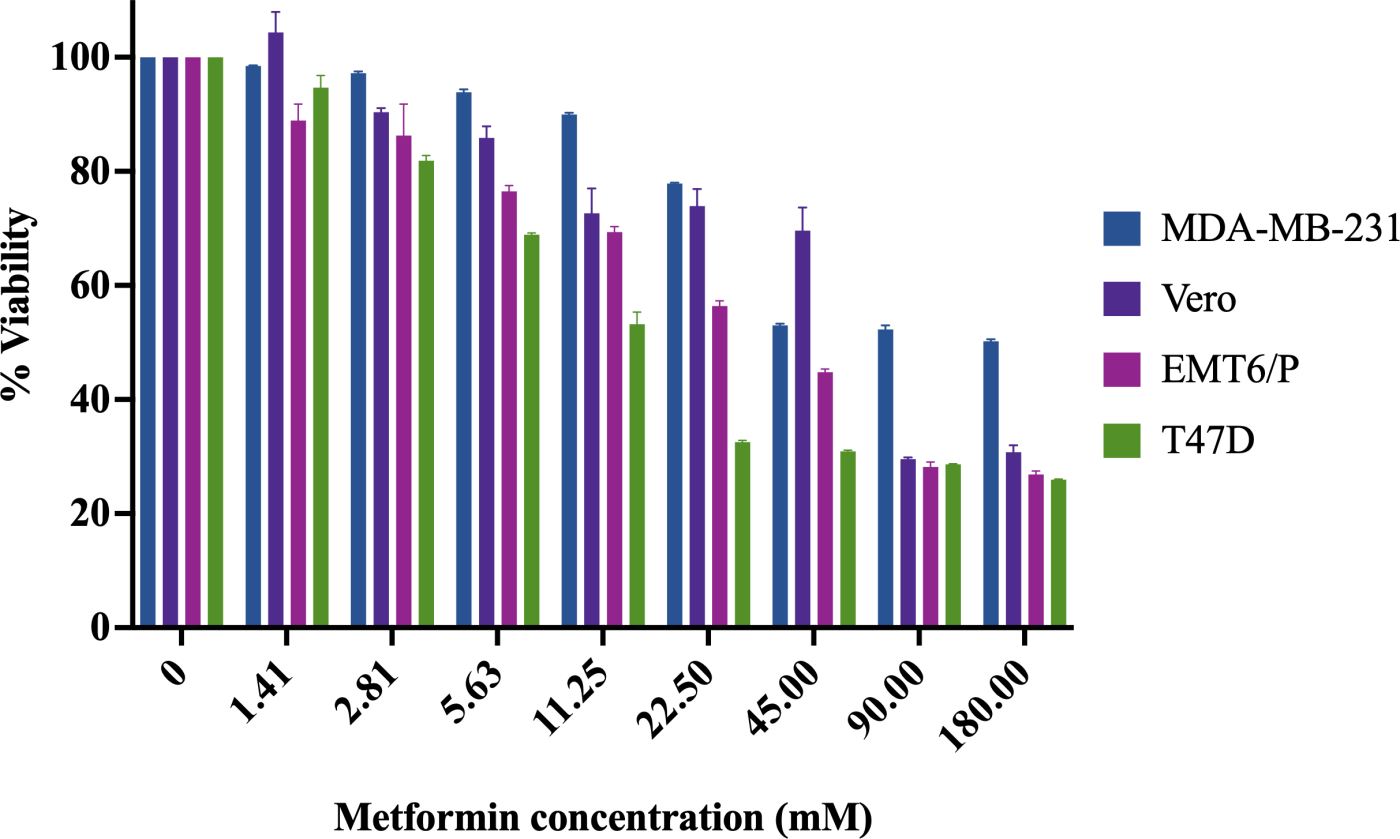
Effect of increasing concentrations of metformin on the viability of different cell lines (MDA-MB-231, Vero, EMT6/P, and T47D).

**Figure 3:**
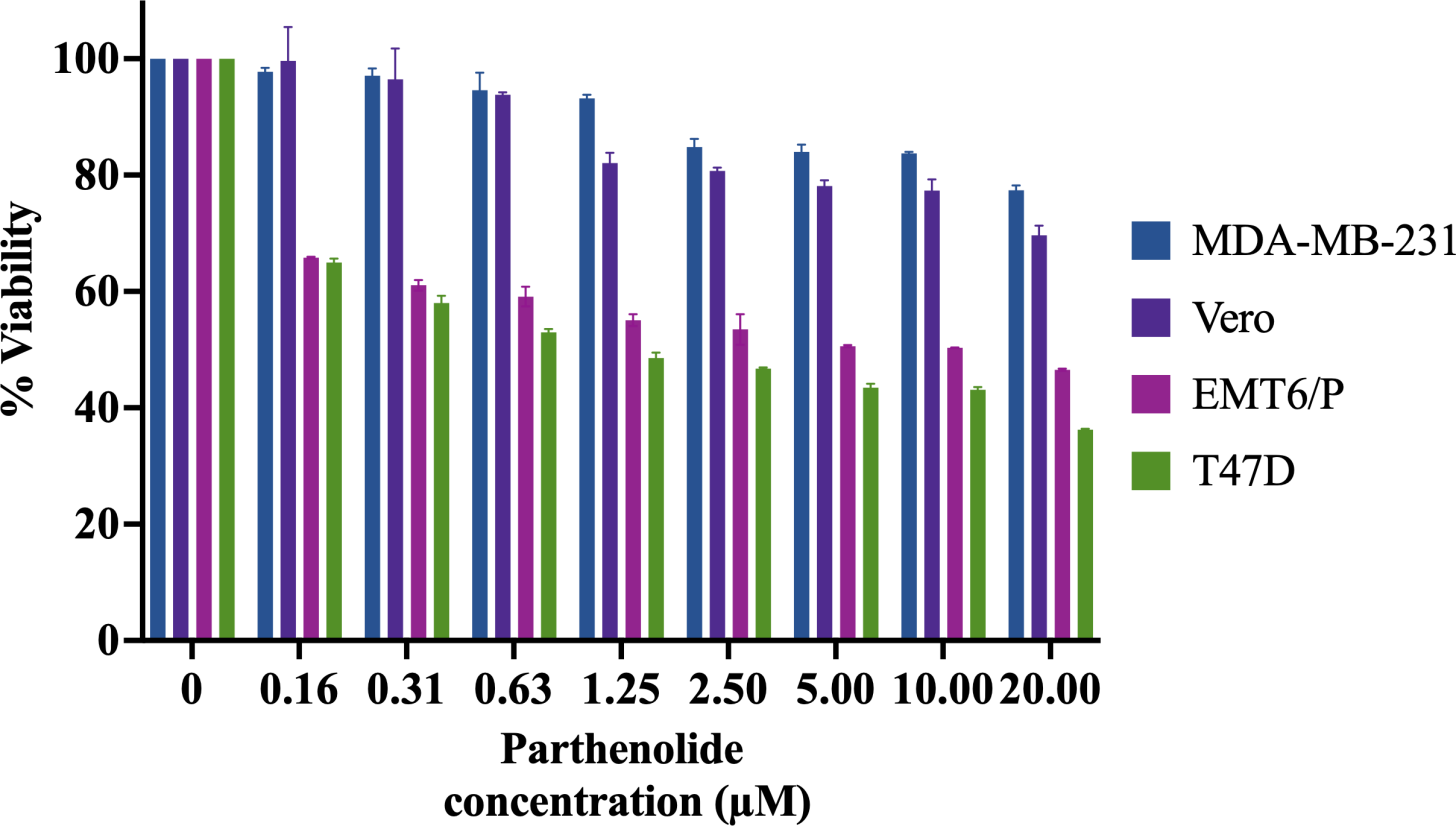
Effect of increasing concentrations of parthenolide on the viability of different cell lines (MDA-MB-231, Vero, EMT6/P, and T47D).

Treatment with increasing concentrations of MET (1.4–180 mM) or PAR (0.16–20 μM) produced a dose-dependent decrease in cell viability across all tested cell lines (**Figures 2**, **3**), with statistical comparisons versus untreated controls summarized in **Tables 2** and **3**. Among the cancer cell lines, T47D cells were the most sensitive to both agents, achieving maximum inhibition rates of 74.03% at 180 mM MET and 63.77% at 20 μM PAR, the highest concentrations tested. This was further supported by the lowest IC_50_ values observed for T47D cells (16.26 mM for MET and 1.72 μM for PAR) (**Table 4**). EMT6/P cells showed slightly lower sensitivity, with maximum inhibition rates of 73.11% and 53.45% at the highest concentrations of MET and PAR, respectively. In contrast, MDA-MB-231 cells retained more than 50% viability even at the highest tested concentrations, indicating reduced susceptibility (**Tables 2**, **3**). IC_50_ values were calculated for all treatments and compared with those of doxorubicin HCl (**Table 4**).

**Table 2:** Antiproliferative activity of increasing concentrations of MET (mM) against different cell lines (MDA-MB-231, Vero, EMT6/P, and T47D).

MET concentration	% Viability			
	MDA-MB-231	Vero	EMT6/P	T47D
1.41	98.46 ± 0.13	104.34 ± 3.63	88.92 ± 2.88 ***	94.68 ± 2.12
2.81	97.21 ± 0.33	90.36 ± 0.73 **	86.26 ± 5.54 ***	81.87 ± 0.92 ***
5.63	93.84 ± 0.53	85.87 ± 2.04 ***	76.50 ± 1.00 ***	68.92 ± 0.28 ***
11.25	89.95 ± 0.33 **	72.67 ± 4.31 ***	69.35 ± 0.94 ***	53.21 ± 2.12 ***
22.50	77.87 ± 0.13 ***	73.91 ± 2.99 ***	56.38 ± 0.94 ***	32.55 ± 0.28 ***
45.00	52.99 ± 0.33 ***	69.59 ± 4.06 ***	44.79 ± 0.55 ***	30.92 ± 0.21 ***
90.00	52.27 ± 0.73 ***	29.55 ± 0.32 ***	28.16 ± 0.89 ***	28.66 ± 0.07 ***
180.00	50.22 ± 0.33 ***	30.79 ± 1.20 ***	26.89 ± 0.61 ***	25.97 ± 0.07 ***

Data are expressed as the mean ± SEM. Statistical significance was determined by comparing each concentration with the untreated control within the corresponding cell line. Significant differences are indicated as **p < 0.01, and ***p < 0.001.

**Table 3:** Antiproliferative activity of increasing concentrations of PAR (μM) against different cell lines (MDA-MB-231, Vero, EMT6/P, and T47D).

PAR concentration	% Viability			
	MDA-MB-231	Vero	EMT6/P	T47D
0.16	97.75 ± 0.68	99.62 ± 5.85	65.81 ± 0.17 ***	64.99 ± 0.68 ***
0.31	97.07 ± 1.24	96.44 ± 5.31	61.06 ± 0.89 ***	58.00 ± 1.26 ***
0.63	94.59 ± 3.04	93.81 ± 0.41	59.12 ± 1.69 ***	52.96 ± 0.58 ***
1.25	93.18 ± 0.62 *	82.06 ± 1.77 ***	55.07 ± 1.01 ***	48.55 ± 0.92 ***
2.50	84.84 ± 1.41 ***	80.70 ± 0.59 ***	53.47 ± 2.61 ***	46.75 ± 0.19 ***
5.00	83.99 ± 1.24 ***	78.12 ± 1.00 ***	50.60 ± 0.17 ***	43.45 ± 0.68 ***
10.00	83.71 ± 0.28 ***	77.34 ± 1.95 ***	50.31 ± 0.04 ***	43.11 ± 0.44 ***
20.00	77.40 ± 0.85 ***	69.63 ± 1.68 ***	46.55 ± 0.17 ***	36.23 ± 0.15 ***

Data are expressed as the mean ± SEM. Statistical significance was determined by comparing each concentration with the untreated control within the corresponding cell line. Significant differences are indicated as *p < 0.05, and ***p < 0.001.

**Table 4:** The IC_50_ values of metformin, parthenolide, their combination, and doxorubicin HCl, and the Combination Index (CI) in EMT6/P, MDA-MB-231, T47D, and Vero cell lines.

Cell line	IC_50_					Combination index (CI)	Interpretation
	MET (mM)	PAR (µM)	MET in combination (mM)	PAR in combination (µM)	Doxorubicin HCl (µM)		
EMT6/P	32.46 ± 0.37	8.8 ± 0.30	18.68 ± 0.54 *	1.64 ± 0.06 *	4.74 ± 0.04	0.869	Slight synergism
MDA-MB-231	181.59 ± 1.08	>20	40.96 ± 0.27 *	9.31 ± 0.37 *	7.35 ± 0.93	0.796	Moderate synergism
T47D	16.26 ± 0.36	1.72 ± 0.12	7.28 ± 0.11 *	0.16 ± 0.01 *	0.87 ± 0.02	0.582	Synergism
Vero	61.19 ± 3.61	>20	>30	>10	29.21 ± 0.16	1.235	Moderate antagonism

Interpretation of results: antagonism for CI value above 1.3; moderate antagonism for CI value of 1.1 to 1.3; additive interaction for CI value of 0.9 to 1.1; slight synergism for CI value of 0.8 to 0.9; moderate synergism for CI value of 0.6 to 0.8; synergism for CI value of 0.4 to 0.6; and strong synergism for CI value of 0.2 to 0.4. Data are expressed as the mean ± SEM. Statistical significance was determined by comparing MET in the combination with MET alone, and PAR in the combination with PAR alone. Significant differences are indicated as *p < 0.05.

**Figure 4:**
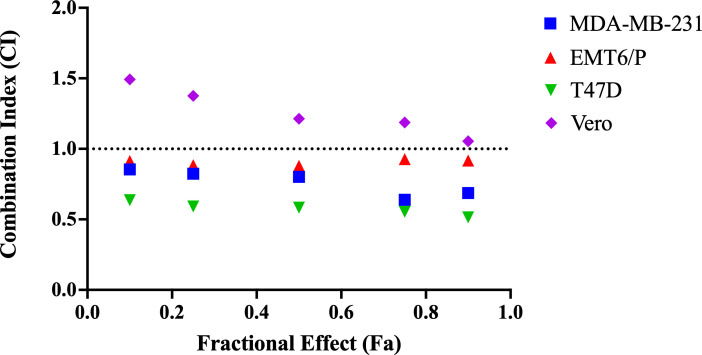
Comparative combination index analysis of metformin and parthenolide across MDA-MB-231, EMT6/P, T47D, and Vero cells.

The combination of MET and PAR exhibited synergistic effects of varying degrees across the cancer cell lines and significantly reduced IC_50_ values compared with individual treatments (*p* < 0.05) (**Table 4**). The strongest synergistic effect was observed in T47D cells, which showed the lowest combination index (0.582) and the lowest IC_50_ values for the combination treatment among all tested cell lines (7.28 mM for MET in combination and 0.16 µM for PAR in combination) (**Table 4**). Comparative combination index analysis further demonstrated differential interaction profiles among the tested cell lines, with consistent synergistic interactions (CI < 1) observed in T47D and MDA-MB-231 cells across the evaluated fractional effect (Fa) range, weaker synergism in EMT6/P cells, and predominantly antagonistic interactions in Vero cells (**Figure 4**).

### Effect on caspase-3 levels in T47D Cells

**Figure 5:**
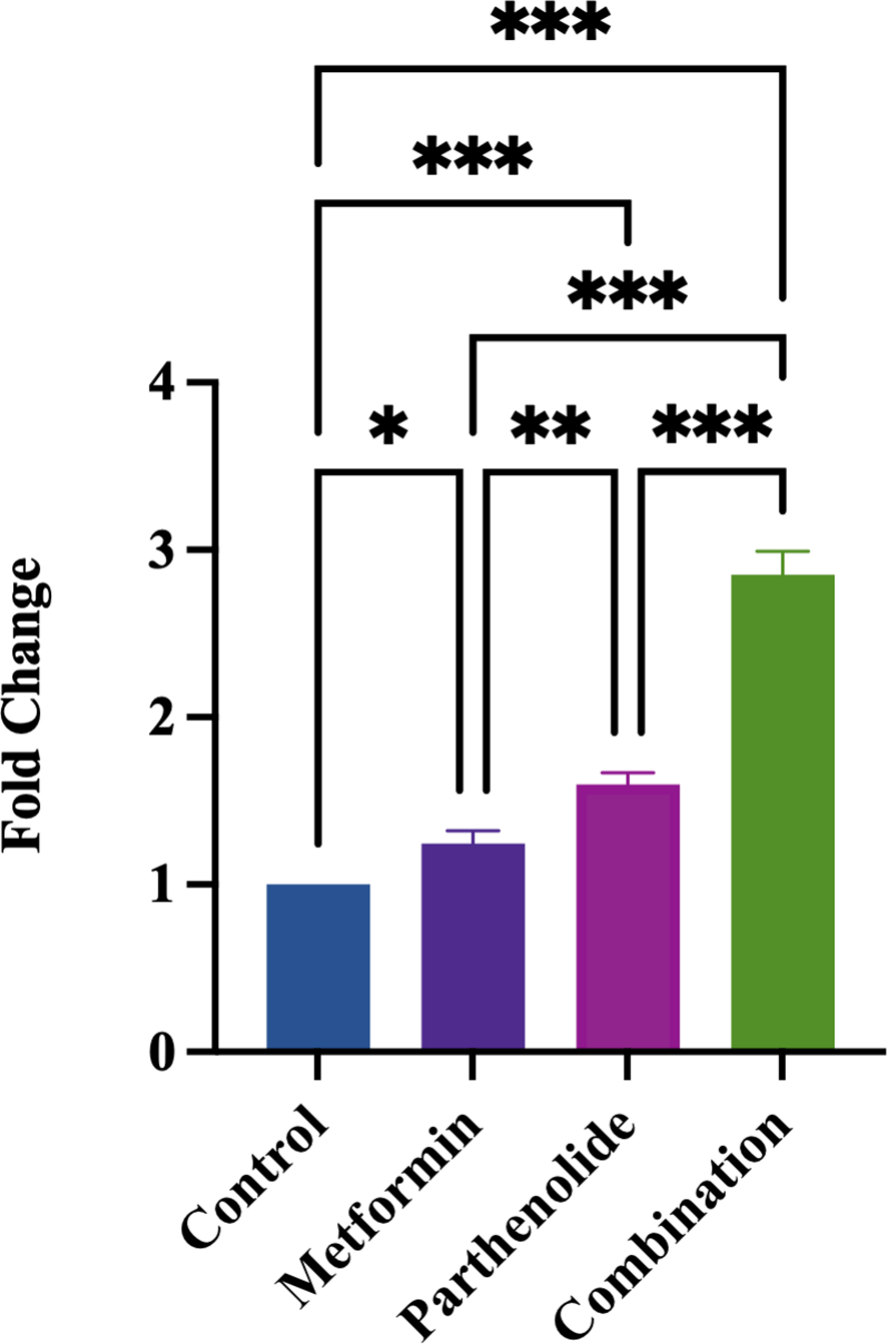
Activity of caspase-3 in T47D cells following treatment with 7 mM MET, 0.2 μM PAR, and their combination (7 mM MET + 0.2 μM PAR). Results are expressed as mean fold change ± SEM (n = 3). Significant differences are indicated as *p < 0.05, **p < 0.01, and ***p < 0.001.

Treatment of T47D cells with MET (7 mM) and PAR (0.2 µM) for 48 hours increased caspase-3 activity compared to untreated control. MET treatment resulted in a moderate elevation in caspase-3 levels (~1.24-fold), while PAR treatment produced a greater increase (~1.60-fold). The combined treatment (7 mM MET + 0.2 μM PAR) yielded the highest caspase-3 activity, reaching approximately 2.85-fold relative to control, and was significantly higher than either treatment alone (**Figure 5**).

### Effect on VEGF expression in T47D cells

**Figure 6:**
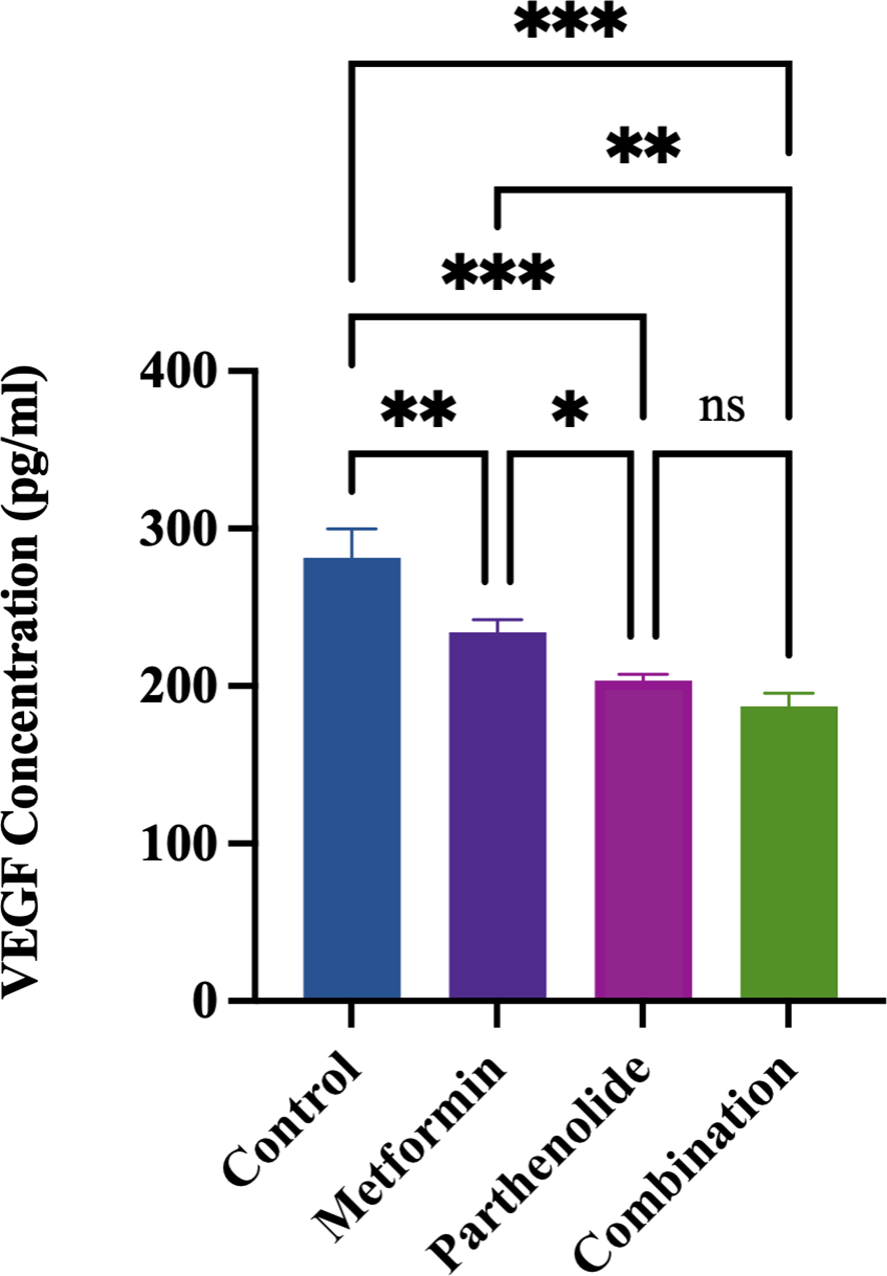
Effect of different treatments on VEGF expression by T47D cells following treatment with 7 mM MET, 0.2 μM PAR, and their combination (7 mM MET + 0.2 μM PAR). Results are expressed as mean VEGF concentration (pg/ml) ± SEM (n = 3). Significant differences are indicated as *p < 0.05, **p < 0.01, and ***p < 0.001. ns, not significant.

The anti-angiogenic potential of the different treatments was evaluated by measuring VEGF expression in cell culture. The untreated control group exhibited the highest VEGF levels (281.63 pg/mL). Single-agent treatment with MET and PAR reduced VEGF levels to 234.01 pg/mL and 203.49 pg/mL, respectively, both representing statistically significant reductions compared to the untreated control (**Figure 6**).

The greatest reduction in VEGF levels was observed with the combination treatment (187.05 pg/mL), which was significantly lower than both the MET-treated and control groups (**Figure 6**).

### Antitumor effect against breast cancer implanted in mice

**Figure 7:**
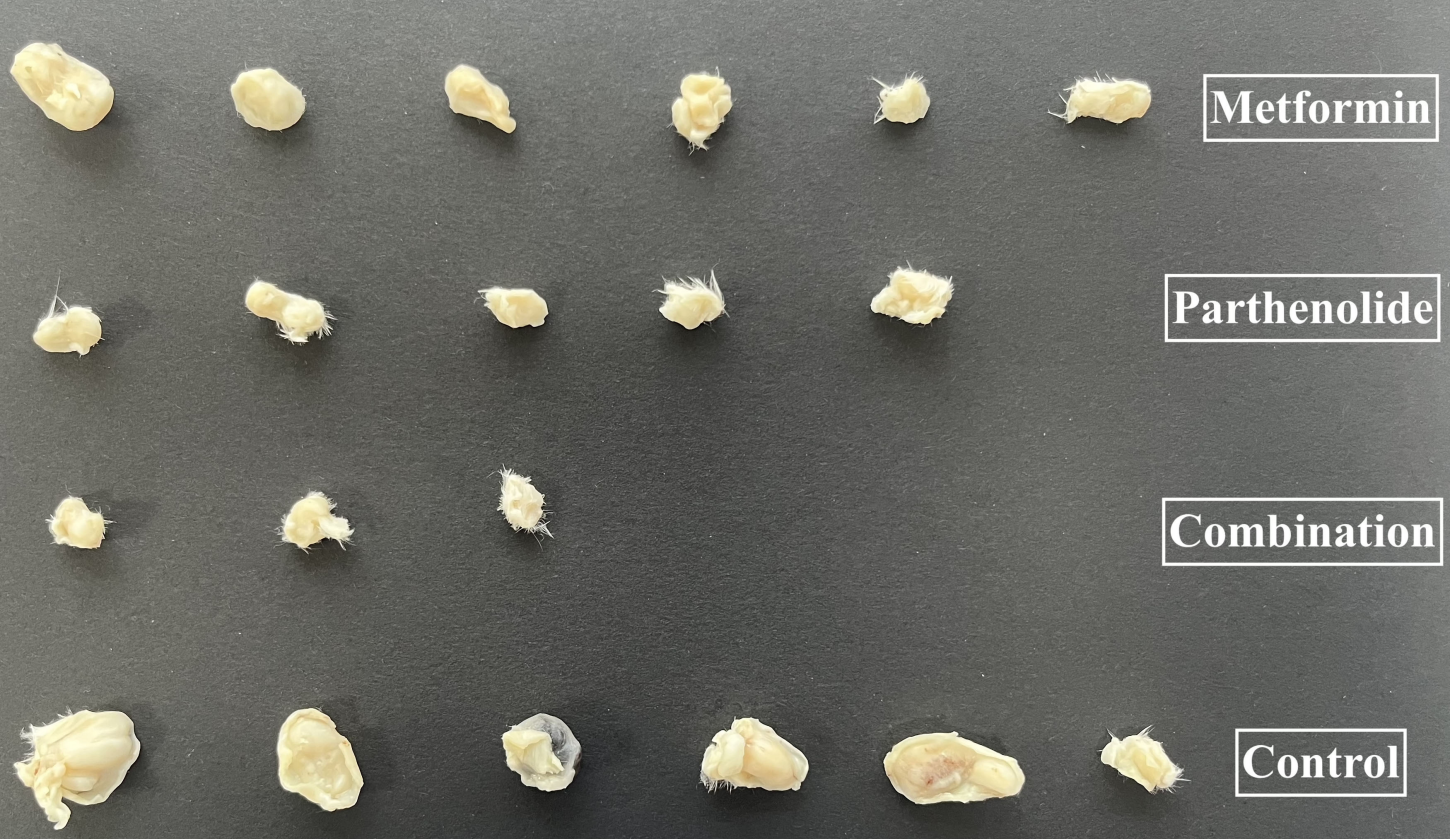
Comparison of final tumor sizes in mice treated with metformin, parthenolide, combination treatment, or control (n = 6). Tumors not shown indicate complete tumor regression. quantitative tumor volume data are presented in **Table 5**.

A significant reduction in tumor size (*p* < 0.05) was observed in mice treated with 80 mg/kg/day MET, showing a percentage change of −23.65%, compared to the untreated control group, which exhibited a 46.15% increase in tumor size. Treatment with 40 mg/kg/day PAR resulted in a greater reduction (−33.25%). The most pronounced decrease was observed in the combination therapy group, with a percentage change of −66.73% (**Table 5**). Furthermore, the highest tumor regression rate (50%) was recorded in the combination group, whereas no cures were observed in the MET group and only 16.67% in the PAR-treated group (**Table 5**). No mortality was reported in any of the treatment groups (**Table 5**). Final tumors collected on day 7 are shown in **Figure 7** to illustrate differences in tumor size among the treatment groups.

**Table 5:** Effects of metformin, parthenolide, and their combination on tumor size (mm^3^), cure rate, and survival.

Treatment	Initial tumor size	Final tumor size	% change in tumor size	% of cured mice	% mortality
Metformin	413.39 ± 44.03	315.62 ± 45.33	-23.65 *	0	0
Parthenolide	398.42 ± 46.55	265.94 ± 49.74	-33.25 *	16.67	0
Combination	402.47 ± 44.89	133.89 ± 12.32	-66.73 * # €	50.00	0
Control	423.98 ± 47.30	619.66 ± 110.27	46.15	0	0

Data are expressed as mean ± SEM (n=6). Statistical significance was defined as *p < 0.05 vs. control, #*p* < 0.05 vs. metformin-treated group, and €*p* < 0.05 vs. parthenolide-treated group. Cure was defined as the absence of detectable tumor at the inoculation site following 7 days of treatment. Mortality (%) was calculated as the proportion of animals that died during the treatment period.

### Effect on serum levels of AST, ALT, and creatinine

To evaluate the effects of the different treatments on liver and kidney function, serum levels of AST, ALT, and creatinine were measured. All treatment groups, as well as the untreated control group, showed no signs of hepatotoxicity or nephrotoxicity, with serum enzyme and creatinine levels remaining within the normal range. These values were compared to a healthy group of mice without tumors. Serum AST, ALT, and creatinine levels in all treatment and control groups were comparable to those of the healthy group, with no statistically significant differences observed (**Table 6**).

**Table 6:** Serum levels of AST, ALT, and creatinine across all experimental groups and the healthy group.

Treatment	AST (U/L)	ALT (U/L)	Creatinine (mg/dl)
Metformin	178.60 ± 10.16	46.33 ± 5.25	0.22 ± 0.03
Parthenolide	207.47 ± 2.38	47.60 ± 4.39	0.17 ± 0.00
Combination	175.20 ± 20.75	51.90 ± 11.21	0.28 ± 0.04
Control	194.67 ± 60.82	43.77 ± 0.55	0.18 ± 0.02
Healthy	153.03 ± 25.13	57.70 ± 7.90	0.16 ± 0.00

Values are expressed as mean ± SEM. No statistically significant differences were observed between the experimental groups and the healthy group (*p* > 0.05).

## Discussion

Metformin (MET) and parthenolide (PAR), the central focus of the present study, have been previously investigated individually and in combination with other therapeutics for their anticancer properties against a broad spectrum of malignancies, including breast cancer (4, 6–9, 14, 18, 27). However, to the best of our knowledge, this is the first study to evaluate the anticancer potential of their combined use as a novel therapeutic strategy. We have evaluated the combination both *in vitro* against a set of breast cancer cell lines (EMT6/P, MDA-MB-231, and T47D) alongside the normal cell line (Vero), and *in vivo* using an EMT6/P-induced breast cancer model in female BALB/c mice. Our findings demonstrated that the combination exerts synergistic inhibitory effects on tumor proliferation both *in vitro* and *in vivo*, mediated through the induction of apoptosis and suppression of angiogenesis. Collectively, these results are promising and were achieved in the absence of any significant toxicity. Importantly, this study was designed as an initial step to evaluate the therapeutic potential of the MET/PAR combination at the functional level, with a focus on key indicators of apoptosis and angiogenesis. While these findings provide a solid foundation, further studies are needed to clarify the underlying molecular mechanisms and validate these effects more comprehensively.

Our *in vitro* cytotoxicity study demonstrated that both MET and PAR inhibited cancer cell proliferation in a dose-dependent manner across all tested cell lines (**Figures 2**, **3**). MET exhibited the greatest antiproliferative effect against T47D cells, yielding the lowest IC_50_ value of 16.26 mM (**Table 4**), which is comparable to previously reported values (28, 29) and consistent with established evidence of MET efficacy against this cell line (30). In contrast, MDA-MB-231 cells were the least responsive to MET treatment among the cancer cell lines tested, possibly due to the well-documented multifactorial resistance mechanisms this cell line is known to adopt (31).

A similar pattern was observed with PAR, where T47D cells again exhibited the greatest sensitivity and MDA-MB-231 cells the lowest. Of particular note during PAR treatment was the behavior of the non-cancerous Vero cell line, which displayed relative resistance with an IC_50_ exceeding 20 µM. This finding aligns with the known selectivity of PAR toward malignant cells, leaving non-cancerous cells largely unaffected (10, 32), and may be considered an indicator of its favorable safety profile *in vitro*.

The combination of MET and PAR significantly reduced IC_50_ values across all tested breast cancer cell lines, with synergistic interactions of varying degrees observed in each. Notably, the strongest synergistic activity was recorded against the T47D cell line (**Table 4**). This was further supported by comparative combination index analysis, which demonstrated sustained synergism across the evaluated fractional effect range in T47D cells, moderate but consistent synergy in MDA-MB-231 cells, weaker interactions approaching additivity in EMT6/P cells, and predominantly antagonistic responses in Vero cells (**Figure 4**). These findings align with a growing body of evidence demonstrating the combinatorial potential of both agents in breast cancer models. MET has previously been shown to act synergistically with a range of anticancer agents, including silibinin (29), melatonin (33), everolimus (34), doxorubicin (35), and rapamycin (36). Similarly, PAR has demonstrated synergistic activity when combined with selinexor (37), epirubicin (38), tamoxifen, mitoxantrone, and vinorelbine (39). Together, these findings suggest that the synergistic activity observed in the present study is not unexpected, given the established combinatorial potential of both MET and PAR in breast cancer.

A relevant observation was that MET displayed a concentration-dependent cytotoxic effect on normal Vero cells, remaining harmless at low concentrations (-4.34% inhibition at 1.41 mM) but reaching approximately 70% inhibition at 90–180 mM (**Table 2**), suggesting a threshold above which toxicity becomes apparent. Interestingly, when MET was used in combination, Vero cell viability (~75.63%) at the first combination concentration point was noticeably higher than that seen with MET alone at its first concentration point, simply because lower concentrations of MET were needed when combined with PAR. These lower concentrations, while still active against cancer cells, remained within the safe range for normal cells. This highlights one of the key advantages of combination therapy: reduced doses, maintained efficacy, and a better safety profile (15).

To further validate the *in vitro* findings, the combination was evaluated *in vivo* and continued to demonstrate promising therapeutic activity, as evidenced by a significant reduction in tumor size (*p* < 0.05) in the combination group compared to both the untreated control and the single-agent groups. This was accompanied by a notable improvement in cure rate, with 50% of animals in the combination group achieving tumor resolution (**Table 5**). Importantly, these outcomes were achieved without any significant hepatic or renal toxicity, as AST, ALT, and creatinine levels in the combination group remained comparable to those of the healthy control group (*p* > 0.05), indicating a favorable hepatic and renal safety profile (**Table 6**). Nevertheless, comprehensive safety profiling, including histopathological evaluation of major organs (liver, kidney, and spleen), would further strengthen the assessment of systemic toxicity and complement the favorable biochemical safety findings observed in the present study.

To gain deeper insight into the mechanisms underlying the anticancer activity of this combination, the induction of apoptosis and inhibition of angiogenesis were investigated. The T47D cell line was selected for this purpose, given its superior responsiveness in the present study and its well-established role as a representative model of the luminal-A breast cancer subtype (40), the most clinically prevalent subtype of breast cancer (41).

Given that caspase-3 is widely recognized as a key executioner of apoptosis and a reliable indicator of apoptotic activity (42), its activation was evaluated across single and combination treatment groups. Both MET and PAR were found to elevate caspase-3 activity as single agents; however, their combination resulted in the most substantial increase in caspase-3 levels among all treatment conditions in T47D cells (**Figure 5**).

These findings are consistent with the established capacity of both agents to induce caspase-3-dependent apoptosis in cancer cells, whether used alone or in combination with other therapeutics (21, 43–45). MET has been widely reported to induce apoptosis in cancer cells, in part through activation of the caspase-dependent pathway, particularly caspase-3, which serves as a key executioner enzyme in programmed cell death (6). Mechanistically, MET activates AMP-activated protein kinase (AMPK) (4, 44), suppresses mTOR signaling (6), and disrupts mitochondrial energy metabolism (4), thereby promoting cellular stress and apoptotic signaling. These effects can enhance mitochondrial membrane permeabilization and trigger cytochrome c release, leading to activation of initiator caspase-9 followed by downstream caspase-3 cleavage and execution of apoptosis (4, 6).

PAR is believed to induce apoptosis by enhancing oxidative stress. The balance between the production of reactive oxygen species (ROS) and the antioxidant systems, including glutathione and thioredoxin, is essential for maintaining intracellular redox homeostasis (11, 14). Research indicates that PAR lowers glutathione levels and inhibits its metabolic enzymes, which leads to an increase in ROS levels initiating the apoptotic pathway (39, 46). Because cancer cells generally experience higher oxidative stress than normal cells, the additional ROS generated by PAR may be sufficient to trigger tumor cell death, whereas normal cells may continue to maintain redox equilibrium through adaptive antioxidant responses. This selective increase in oxidative stress may partly explain the ability of PAR to target cancer cells for cell death while sparing normal cells (46).

Finally, the ability of the combination to inhibit angiogenesis was assessed through measurement of VEGF expression across all treatment groups. As anticipated, the untreated control exhibited the highest VEGF levels, while both MET and PAR individually produced significant reductions in VEGF expression, which is an effect that has been widely reported in the literature (47, 48).

MET has been associated with reduced microvessel density, potentially due to decreased signaling through platelet-derived growth factor B (PDGF-B) and its receptor PDGF-Rβ (4). Additionally, MET leads to reduced endothelial function and lower levels of vascular inflammation mediators, including VEGF, plasminogen activator inhibitor-1 (PAI-1), hypoxia-inducible factor 1-alpha (HIF-1), and von Willebrand factor, likely through the inhibition of mTOR signaling (49). These mechanisms collectively contribute to the anti-angiogenic effects of MET. Wang et al. reported that MET inhibited lung metastasis of breast cancer by suppressing angiogenesis and promoting vessel normalization (50).

In many cancer types, NF-κB is constitutively active, regulating genes essential for tumor growth and angiogenesis (39). PAR, at pharmacologically relevant, noncytotoxic concentrations (1–10 μM), has been shown to inhibit NF-κB through multiple mechanisms, both directly and indirectly. Two primary mechanisms have been identified: direct interaction of PAR with NF-κB subunits, particularly at lower doses, and inhibition of the IκB kinase (IKK) complex at higher doses (12, 14).

In the present study, co-treatment with MET and PAR produced the most pronounced VEGF suppression compared with either agent alone, suggesting that concurrent administration may enhance the anti-angiogenic activity of both compounds.

Taken together, the observed modulation of both apoptotic and angiogenic pathways suggests that the MET/PAR combination may influence multiple molecular targets. Given that the present study evaluates a combination of two agents, the observed synergistic effect is likely mediated through interactions with distinct and potentially complementary targets rather than a single defined protein. Further molecular and pathway-level characterization will therefore be essential to delineate the specific proteins involved. Within this framework, future integration of computational modeling approaches, including molecular docking and molecular dynamics simulations, may offer valuable insight into potential ligand–target interactions and support a more comprehensive mechanistic understanding.

Despite the encouraging findings, several limitations of the present study should be acknowledged. The study was designed as an initial investigation focusing primarily on functional outcomes related to apoptosis and angiogenesis; therefore, in-depth molecular characterization was beyond its scope. In addition, only a limited number of breast cancer models were evaluated, which may restrict the generalizability of the findings across the full heterogeneity of breast cancer subtypes. Furthermore, pharmacokinetic interactions between MET and PAR, as well as the bioavailability of PAR, were not evaluated, which are important for comprehensive translational evaluation of this combination.

## Conclusion

In conclusion, MET and PAR worked synergistically against breast cancer both *in vitro* and *in vivo*, with apoptosis induction and angiogenesis inhibition playing a role in this effect. Importantly, these effects were achieved without any significant impact on hepatic or renal function. Both agents are widely available and relatively affordable, which makes this combination a particularly attractive candidate for further clinical investigation in breast cancer.

Notably, the present study represents an initial step, and much remains to be understood regarding the full scope of mechanisms underlying this synergy. Future studies should aim to provide a more comprehensive mechanistic and translational characterization of the MET/PAR combination. This includes tumor tissue-level analysis of caspase-3 and VEGF expression to further validate the *in vivo* findings, as well as investigation of upstream signaling pathways such as PI3K/Akt, NF-κB, and mitogen-activated protein kinase (MAPK), together with protein-level validation using Western blot analysis. Additional functional assays, including ROS generation, Annexin V staining, mitochondrial membrane potential assessment, and gene expression profiling, would further strengthen mechanistic insight.

## Data Availability

The original contributions presented in the study are included in the article/supplementary material. Further inquiries can be directed to the corresponding author.
